# Density-functional-theory simulations of the water and ice adhesion on silicene quantum dots

**DOI:** 10.1038/s41598-022-11943-w

**Published:** 2022-05-20

**Authors:** Tianpei Duan, Wei Wu, Kwang-Leong Choy

**Affiliations:** UCL Institute for Materials Discovery, University College London, Malet Place, London, WC1E 7JE UK

**Keywords:** Materials for devices, Sensors and biosensors, Materials science, Nanoscale materials, Quantum dots, Two-dimensional materials

## Abstract

The absorption of water and ice on silicon is important to understand for many applications and safety concerns for electronic devices as most of them are fabricated using silicon. Meanwhile, recently silicene nanostructures have attracted much attention due to their potential applications in electronic devices such as gas or humidity sensors. However, for the moment, the theoretical study of the interaction between water molecules and silicene nanostructures is still rare although there is already theoretical work on the effect of water molecules on the silicene periodic structure. The specific conditions such as the finite size effect, the edge saturation of the silicene nanostructure, and the distance between the water/ice and the silicene at the initial onset of the contact have not been carefully considered before. Here we have modelled the absorption of a water molecule and a square ice on the silicene nanodot by using hybrid-exchange density-functional theory, complemented by the Van der Waals forces correction. Three different sizes of silicene nanodots have been chosen for simulations, namely $3\times 3$, $4\times 4$, and $5\times 5$, with and without the hydrogen saturation on the edge. Our calculations suggest that the silicene nanodots chosen here are both hydrophilic and ice-philic. The water molecule and the square ice have tilted angles towards the silicene nanodot plane at ~ 70º and ~ 45º, respectively, which could be owing to the zig–zag structure on silicene. The absorption energies are size dependent for unsaturated silicene nanodots, whereas almost size independent for the hydrogen saturated cases. Our work on the single water molecule absorption energy on silicene nanodots is qualitatively in agreement with the previous theoretical and experimental work. However, the ice structure on silicene is yet to be validated by the relevant experiments. Our calculation results not only further complement the current paucity of water-to-silicene-nanostructure contact mechanisms, but also lead to the first study of square-ice contact mechanisms for silicene. Our findings presented here could be useful for the future design of semiconducting devices based on silicene nanostructures, especially in the humid and low-temperature environments.

## Introduction

Nowadays electronic devices are ubiquitous and indispensable for our daily life. The lifetime and reliability of the electronic devices are important both for the device functionalities and the environmental control. The water intervention to the electronic devices is an important aspect for maintaining and enhancing the device performance^1,2^ because this type of phenomenon can usually lead to a series of safety issues, sometimes even catastrophes. As silicon is the cornerstone for electronic devices, the performance of most of the electronic devices can be deteriorated by the moisture/steam/ice on silicon inevitably in many scenarios even with the device insulation in place, thus causing short-circuiting, reduced sensitivity or even failure of sensors, such as those used in high-temperature production environments or polar developments. The current research in this area is to avoid electronic device failure utilising airtight isolating layers rather than exploring the underlying mechanisms of the hydrophobicity/hydrophilicity of electronic devices^1^. Studying the interaction between the water molecule and the electronic device is crucial to solve the problem fundamentally and identify the optimal materials to prevent the water intervention. On the other hand, the study of this type of interaction can be useful for the design of advanced humidity sensors with high sensitivities.

As the counterpart to graphene, silicene has been progressively researched and developed in the recent years. The difference of silicene from graphene is that silicene has a zig–zag structure^3^, which can provide energetically different reaction sites and brings forward complexity and new possibilities for physical or chemical interactions. Especially, the band gap of the silicene could be tuned (up to 0.5 eV) by chemically introducing sodium atoms^4^, which is a clear advantage compared with graphene that almost has no band gap except for multi-layer structures, and particularly promising for developing semiconducting devices. Silicene holds many physical properties that are comparable with graphene, such as high carrier mobility^5–7^, anomalous integer quantum Hall effect^8–12^, giant magnetoresistance^13–16^ and superconductivity^17–19^. However, as a promising material for electronic devices such as gas sensors^20–22^, field effect transistors^15,23,24^, photodetectors^25–27^ and biodegradable drug carriers^28^, the studies of the surface wetting properties of silicene and its nanostructures are still rare^29^. Furthermore, the conventional waterproofing techniques are challenging to be implemented in the real-world applications^30–32^. silicene nanodots (SND) or nanoflakes have been shown to have more amenable reaction sites at the nanoscale than graphene in the same form^33–35^, which is associated with their higher chemisorption ability^25,26^. Nevertheless, this higher ability may imply that silicenes would absorb water molecules to a greater extent than graphene^36^, which might be detrimental for many further electronic applications of silicenes.

The theory for the properties of silicene has also been developed rapidly in recent years. The electronic structure (such as the band gap) and the mechanical properties of silicenes have been computed from first principles, as reviewed in Ref.^37^. Therein, the simulations for the functionalization of silicene by using hydrogen or florine, to engineer the band gap, have also been summarized. Previously silicene and its interaction with external fields and the other species such as water molecules have been studied theoretically. Drummond, et al., have demonstrated theoretically that the band gap of silicene can be tuned by using electrical fields^5^. To further control the band gap, the other research groups have proposed to make silicene nanomesh (making holes on silicenes)^38^ and absorb appropriate species such as alkali^39^. Hu, et al., have performed first-principles density functional theory (DFT) calculations on the different number of water molecule absorbed by the periodic silicene^40^. Their research has showed that the number of water molecules absorbed has a significant effect on the behaviour of the absorption. The substrates for silicene are also important for its practical application in electronic devices. Chen et al., have modelled the effects of a variety of substrates on the silicene and found Al-terminated Al_2_O_3_(0001) might be a good choice^24^.

In addition, the absorption of a water molecule and ice structures on graphene nanostructure has been studied previously to assess the performance of the current density-functional approximation^41^. The authors therein have found that DFT approximations with London dispersion forces can provide great results. BLYP-D4, TPSS-D4, rev-vdW-DF2, and PBE0-D4 have the best performance among the tested methods. Notice that most of the carbon nanostructures studied are hydrophilic except the benzene that can be hydrophobic for the zero-leg configuration (the water molecule stands on the benzene ring with oxygen nearer to the ring). In addition, the water absorption on the graphite and graphene has been studied by the Van der Waals-force-corrected DFT, which is a very promising method without using any fitting parameters^42^. Moreover, the absorption of water molecule clusters on the graphene has been modelled from first principles, which showed the similar results for the absorption energies^43^. The calculations mentioned above showed the graphene was hydrophilic with the absorption energies in the order of 10 meV, which are much smaller than those for SND^40^.

To the authors’ best knowledge, the detailed study of the absorption of water molecules on silicene nanostructures such as SND is still absent. In particular, the theoretical model of the variation of the surface properties of the silicene due to the absorption of water molecules is needed to understand the behaviour of the water molecule on silicene, hence facilitating the practical design of the future silicene-based electronic device. It is therefore necessary to use suitable computational modelling methods to reveal the microscopic picture for the interaction between the SND and water molecules. Additionally, the application of the silicene-based electronic devices in the low-temperature environment is going to be a challenging task. Considering that the working capacity of electronic devices such as graphene-based gas detectors is reduced below room temperature^44^ and that similar low-temperature capabilities of silicene as a comparator have rarely been investigated, our study explores the interaction of silicene with the low-temperature state of water, i.e. ice, in the scenario where the silicene is used in the electronic devices under the extreme weather condition.

In this work, by using the hybrid-exchange DFT, complimented by the dispersion forces^45^, we have modelled from first principles the interactions between the water molecules and the SND in different attaching positions and with different SND sizes. Two types of SNDs (with and without the hydrogen saturation on the edge) have been investigated. The absorption energies and the effects of the water molecule and the square ice on the vibrational frequencies have been analysed in detail. We demonstrate that SNDs generally possess hydrophilic or ice-philic properties, mainly owing to the formation of the chemical bonds between the silicon and the oxygen atoms. The remaining discussions fall into three sections. In the section II, we introduce our computational methods. In the section III, we present our calculation results. In the section IV, we draw some general conclusions.

## Computational methods

**Figure 1:**
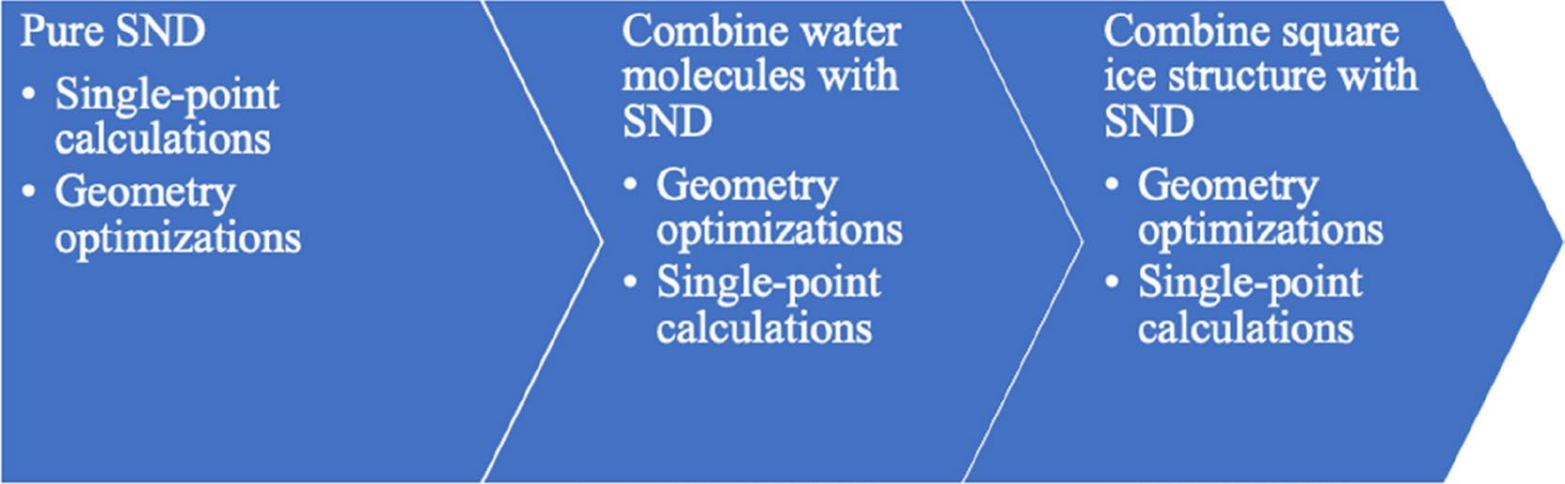
The simulation process for the pure SND and the absorption of a water molecule and a square ice on the SND.

**Figure 2:**
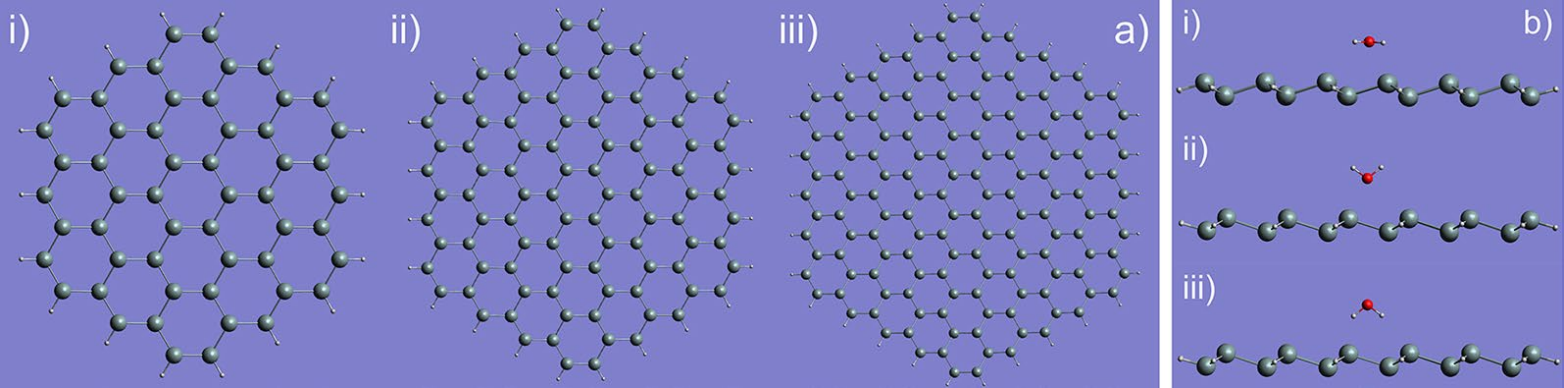
(**a**) The initial structures for the SNDs with different sizes. (i) $3\times 3$, (ii) $4\times 4$; and (iii) $5\times 5$. (**b**) The water molecules with three different initial orientations were put on the $3\times 3$ SND (and the other two SNDs as well, not shown here). (i) is the lying-down configuration, (ii) standing-up (zero-leg), and (iii) standing-up (two-leg). The different distances of 1, 2, and 3 angstroms (near, moderate, and far) were considered, which are close to the Si–O bond length (~ 1.5 angstroms).

As shown in Fig. 1, in our simulations we have first performed single-point calculations and geometry optimizations for the SND, followed by those for the absorption of the water/ice on SND. The silicene lattice constant *a* for a hexagonal lattice was initially set as 3.82 Å with a distance of $\Delta Z$ = 0.44 Å between the top and the bottom layers of silicon atoms in SND. We have presented three hydrogen-saturated SND structures (namely $3\times 3$, $4\times 4$, and $5\times 5$) in Fig. 2 for illustrations.

The geometrical optimizations of the SNDs have been performed to see the finite-size effect and the restructuring on the edge. Two different structures (with and without the hydrogen saturation) were calculated using the B3LYP hybrid-exchange density functional^46^ and the 6–31 g^47^ and CEP-4G^48,49^ basis sets, implemented in Gaussian 09 code^50^. This method was complimented by the calculations taking into account the dispersion forces^45^ for comparison. The absorption energy of the water molecule onto the silicene is defined by the following Eq.^40,42,51^:${E}_{a}={E}_{\mathrm{SND}-\mathrm{D}}-{E}_{\mathrm{D}}-{E}_{\mathrm{SND}}$, where $D$ is a water molecule or a square ice. Here ${E}_{\mathrm{SND}-\mathrm{D}}$ labels the total energy of the optimized combined structure, while ${E}_{\mathrm{D}}$ and ${E}_{\mathrm{SND}}$ refer to the total energies of the water molecule/square ice and the SND alone, respectively. The water molecules and the square ice (we have adopted its structure in Ref.^43^) have been absorbed at the centre and the edge of the SND. We have also compared the infrared (IR) spectra and vibrational frequencies of the combined structures and SNDs, to identify the signature of the water molecule^32,52–56^. The silicon atom is depicted in grey, oxygen in red, and hydrogen in white throughout the paper. The colour coding for the molecular orbitals, such as the highest occupied molecular orbital (HOMO) and the lowest unoccupied molecular orbital (LUMO), is that the positive value is depicted in red and negative in blue.

## Results and discussion

**Figure 3:**
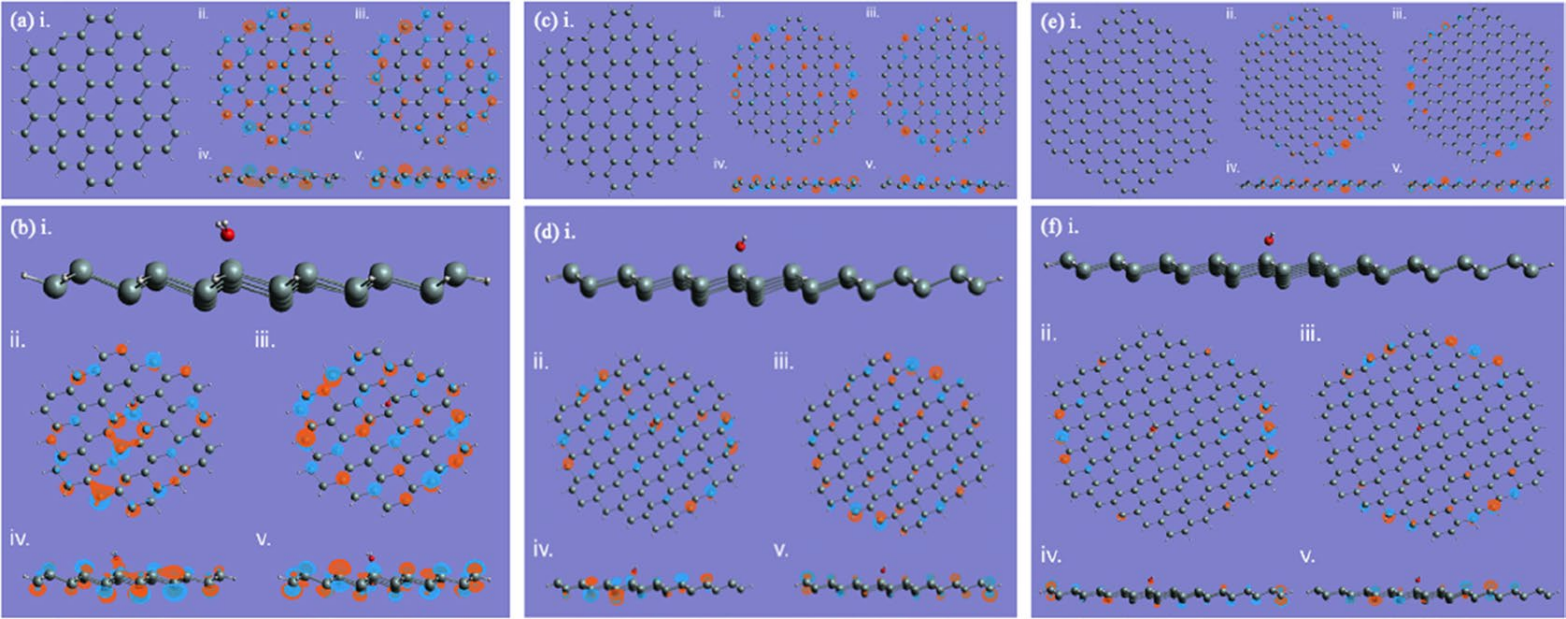
We show the water-molecule absorptions on the $3\times 3$ SND (**a**,**b**), the $4\times 4$ (**c**,**d**), and the $5\times 5$ (**e**,**f**). The optimized structures without and with the water molecule are shown in (i) for each scenario, respectively. In each sub-figure, (ii) and (iii) ((iv) and (v)) are the top (side) views of HOMO and LUMO, respectively.

In Fig. 3, we show the calculation results for the SND without (a, c, e) and with (b, d, f) a water molecule absorbed with an initial zero-leg or two-leg geometries. The calculation results for the $3\times 3$ SND is in (a) and (b), $4\times 4$ in (c) and (d), and $5\times 5$ in (e) and (f). In each sub-figure of Fig. 3, we show the optimized geometry in (i), the top (side) views of HOMO and LUMO in (ii) and (iii) ((iv) and (v)), respectively. After the geometry optimization, all the structures computed here end up with so-called the zero-leg configuration, where the oxygen atom of the water molecule is closer to the SND than the hydrogen atoms. The HOMO and LUMO for the $3\times 3$ SND are shown in Fig. 3a,b as well. From the charge distributions, we can see that the interaction between the water molecule and SND is weak. Similarly, for $4\times 4$ and $5\times 5$, all the water configurations on SNDs end with the zero-leg, as shown in Fig. 3c–f, respectively. We can also notice that for all the three scenarios, the water molecular plane is tilted on the SND surface at ~ 70° (not perpendicular). This may be attributed to the presence of several different reactive sites in SND^57^ due to its zig-zag structure, and the combined effects among the sites result in this slightly skewed orientation of the water molecule. In addition, the HOMO and LUMO are dominated by the 3*p* orbitals on the silicon sites, forming a conjugated system although the structure is not purely two-dimensional. As the size of the SNDs increases, we can see that the charge distribution tends to be more localized on the edge. This may have some effects on the water molecule absorption, e.g. the water molecule will be attached more easily on the edge than in the centre, as can be proved in the calculation results suggested later on in Table 1.

**Table 1:** The optimized water/square-ice-SND distance and absorption energies are shown for all the interaction scenarios considered here.

Interaction scenarios between water molecule, square ice and SNDs							
Silicene	Position and basis set					*d*/Å	${E}_{a}$/ eV
Unsaturated SND (3 × 3)	Lying down (6–31 g)			Inside	Centre	2.122	− 0.27
					Edge	1.993	− 1.09
				Outside	Centre	2.051	− 0.66
					Edge	1.993	− 1.09
	Standing up (6–31 g)		Zero-leg	Inside	Centre	2.170	− 0.28
					Edge	1.993	− 1.09
				Outside	Centre	2.051	− 0.66
					Edge	1.993	− 1.09
			Two-leg	Inside	Centre	2.060	− 0.52
					Edge	1.993	− 1.09
				Outside	Centre	2.051	− 0.66
					Edge	1.993	− 1.09
	Square ice (6–31 g)			Inside	Centre	1.884	− 0.99
					Edge	1.871	− 1.70
				Outside	Centre	1.886	− 0.27
					Edge	1.873	− 1.78
Saturated SND	Lying-down water (CEP-4G)		Centre	$3\times 3$		2.279	− 0.33
				$4\times 4$		2.274	− 0.35
				$5\times 5$		2.272	− 0.34
			Edge	$3\times 3$		2.241	− 0.51
				$4\times 4$		2.235	− 0.53
				$5\times 5$		2.231	− 0.53
	Standing water (CEP-4G)	Zero-leg	Centre	$3\times 3$		2.278	− 0.33
				$4\times 4$		2.274	− 0.35
				$5\times 5$		2.270	− 0.34
			Edge	$3\times 3$		2.241	− 0.51
				$4\times 4$		2.235	− 0.53
				$5\times 5$		2.231	− 0.53
		Two-leg	Centre	$3\times 3$		2.278	− 0.33
				$4\times 4$		2.274	− 0.35
				$5\times 5$		2.272	− 0.34
			Edge	$3\times 3$		2.241	− 0.51
				$4\times 4$		2.235	− 0.53
				$5\times 5$		2.231	− 0.53
	Square ice (CEP-4G)		Centre	$3\times 3$		2.030	− 0.99
				$4\times 4$		2.029	− 1.00
				$5\times 5$		2.027	− 1.01
			Edge	$5\times 5$	Near	2.027	− 1.20
					Moderate	2.058	− 0.86
					Far	2.027	− 1.20

**Figure 4:**
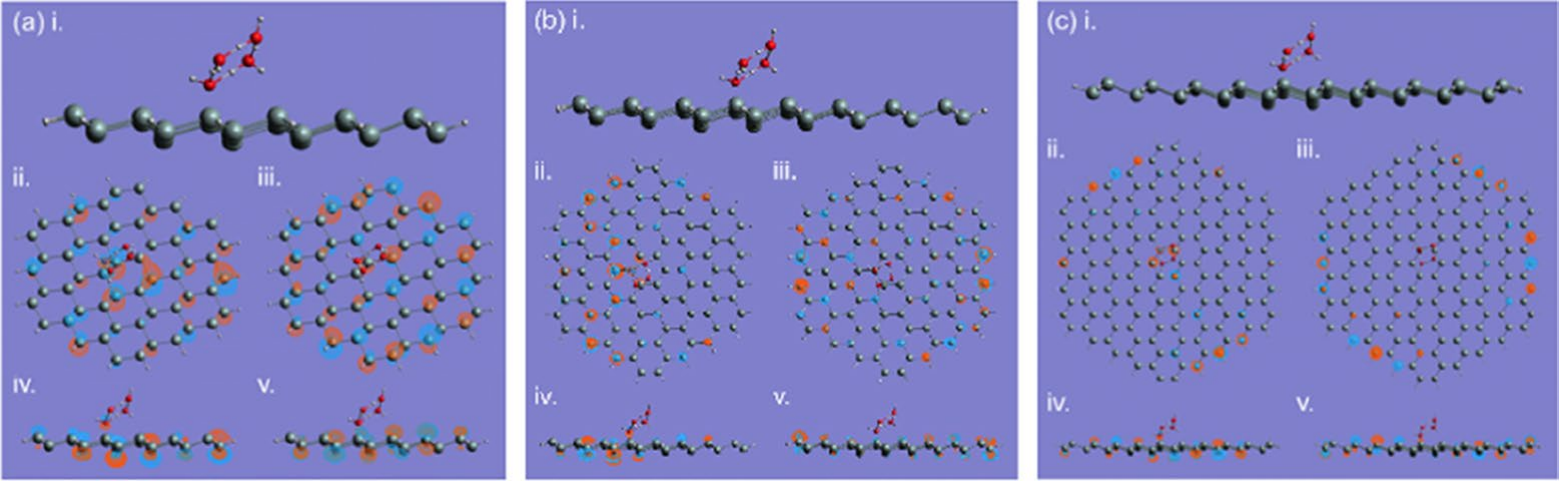
The square ice absorptions on the $3\times 3$ , $4\times 4$ , and $5\times 5$ SND are shown in (**a**–**c**), respectively. In each sub-figure, (i) is the optimized structure, (ii) and (iii) ((iv) and (v)) are the top (side) views of HOMO and LUMO, respectively.

For the absorption of the square ice on the SNDs (Fig. 4), we can see the similar behaviour, i.e., the square ice plane is tilted towards the SND plane, but the titling angle is smaller than the water molecule, which is ~ 45°. Another complication of the square ice absorption compared with the water molecule is the hydrogen atom movement. The hydrogen atoms that are initially coplanar with the oxygen atoms, are now out of the square ice plane, owing to the interaction with the SNDs. This suggests the silicon-oxygen bond formation will push further the hydrogen atoms of the water molecule. The square ice might be dissociated due to the interaction with SND^40^. However, in our calculations, we are not able to identify the complete dissociation of the square ice, which is only distorted or stretched and maintains its original shape. This might be due to the hydrogen bonds in the square ice^58^, which can stabilize the structure. However, there might be some attaching position and water–silicon distances that may cause the complete dissociation, which can be studied in the future.

**Figure 5:**
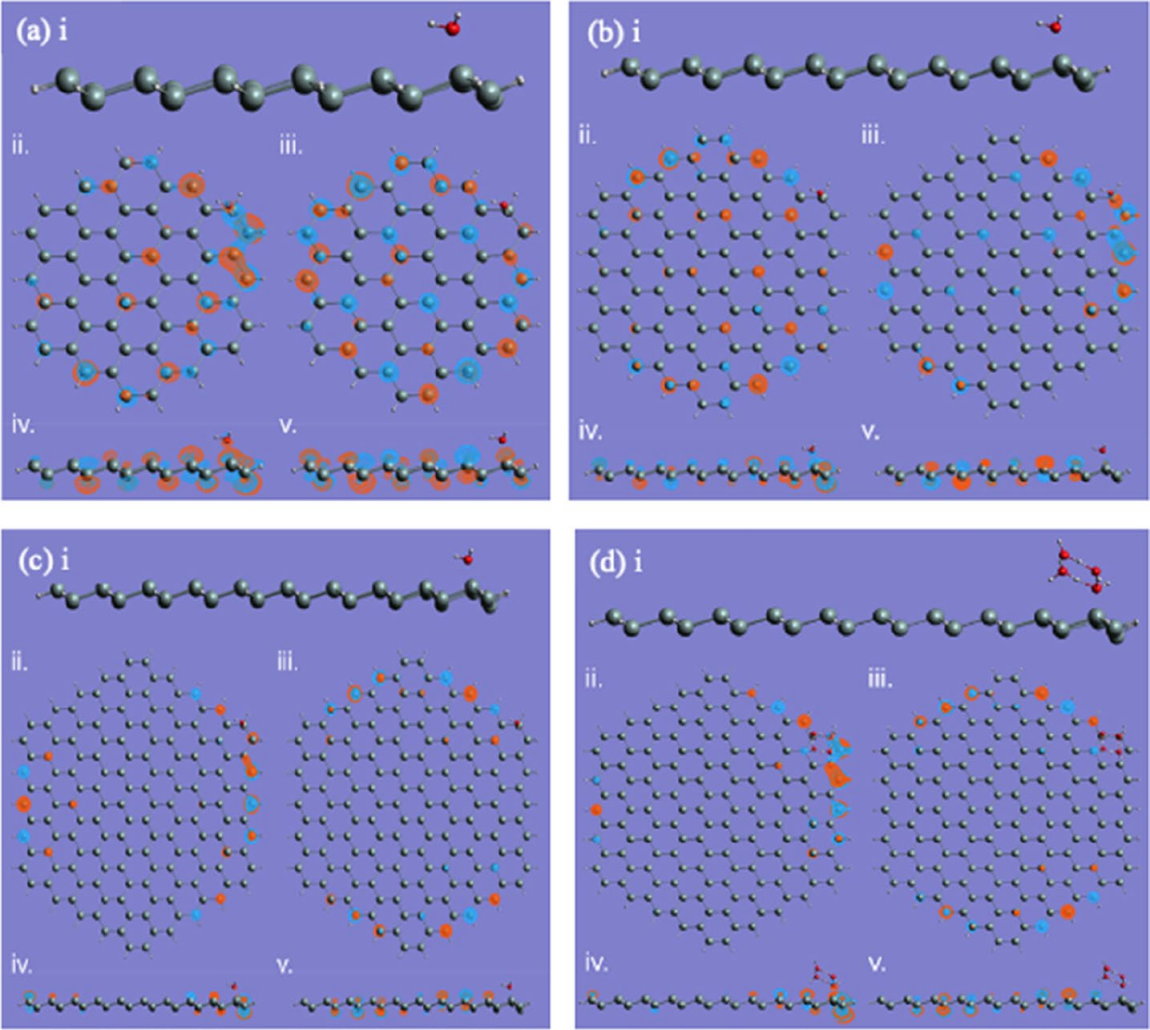
The water-molecule absorptions on the edge of the SND: (**a**) $3\times 3$ ; (**b**) $4\times$ 4; (**c**) $5\times 5$. The ice square on $5\times 5$ is shown in (**d**). The optimized structures are shown in (i) for each scenario, respectively. In each sub-figure, ((ii) and (iii)), and ((iv) and (v)) are the top (side) views of HOMO and LUMO, respectively.

In Fig. 5 we show the water molecule absorption on the edge of SND, where the final contact results are very similar to those at the centre as shown in Fig. 3. This suggests that SND has a stable mechanism of contact with water molecules that does not shift with the reaction site or the size of the SND structure. Figure 5d illustrates the situation where the square ice comes into contact with the edge of the SND. Similar to what happens at the centre, the silicon atom in direct contact with that oxygen atom in the ice structure responsible for forming the contact is slightly depressed, with the final bond length stabilised at 2.027 Angstroms. This finding demonstrates the stability of the silicene and succinctly establishes that the square ice does have some specific contact angles and symmetries on the SND, both at the centre and at the edges, as shown in Figs. 4 and 5.

**Figure 6:**
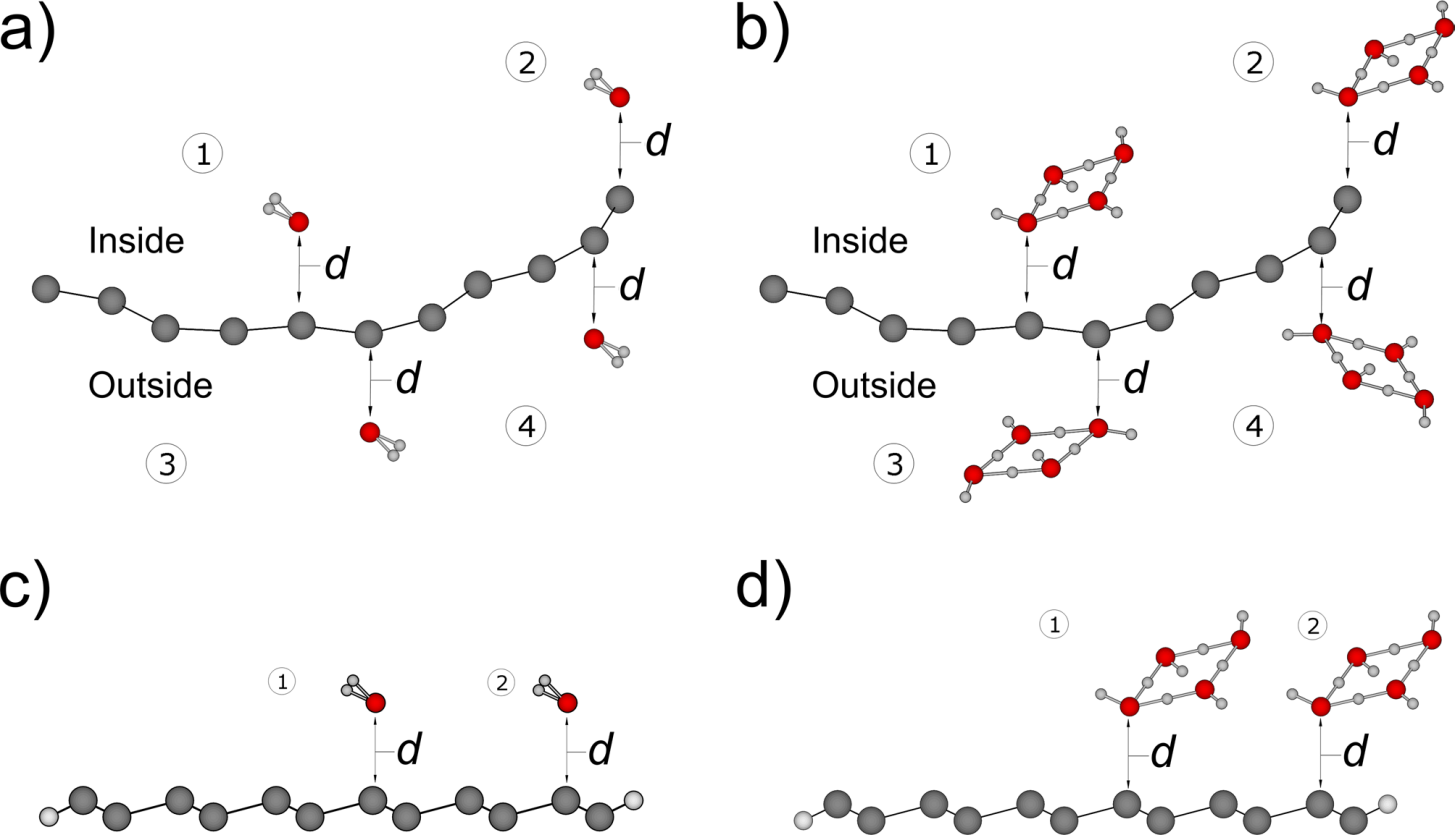
The stable adsorption scenarios of a water molecule and a square ice on the SND. (**a**) a water molecule adsorbed inside and outside the unsaturated SND with a curvature, at the centre and the edge: ① inside and centre, ② inside and edge, ③ outside and centre, and ④ outside and edge. (**b**) a square ice adsorption: ① inside and centre, ② inside and edge, ③ outside and centre, and ④ outside and edge. (**c**) a water molecule adsorbed at the centre and edge of the saturated SND: ① centre, ② edge. (**d**) a square ice adsorption: ① centre, ② edge. The stable bond lengths *d* are calculated as shown in Table 1. The calculations based on various initial spacings (1, 2, and 3 angstroms) are applied to each adsorption scenarios and result in stable and equivalent adsorption distances *d* for each.

To better illustrate the various absorption scenarios recorded in the table, we present Fig. 6 to correspond with the Table 1. The inside/outside refers to the separate studies of the adsorption scenarios inside and outside the curved surface when the silicene is slightly bent without the edge hydrogen saturation. The (a) and (b) represent the water molecule/ square ice adsorption studies on the unsaturated silicene. (c, d) are the same contact studies on the hydrogen saturated silicene. The measurement of "*d*" is determined by taking the distance between the water or ice bonding atom and the silicon atom responsible for the bonding on the silicene during adsorption.

As shown in Table 1, the absorption energies have been calculated for unsaturated and saturated SNDs for a water molecule and a square ice. All the energies are negative, which means all the SNDs here are hydrophilic and ice-philic. For the unsaturated SNDs, the absorption energies for the edge are larger than the centre. This also applies to the square ice. For the saturated SNDs, the absorption energies of water molecules at the centre are ~ 0.3 eV, which is smaller than those at the edge (~ 0.5 eV). For the square ice the absorption energies are ~ 1 eV both for the centre and the edge absorptions. However, by average, the absorption energies of the square ice are larger at the edge than those at the centre, which is consistent with the water absorption calculations. Comparing unsaturated and saturated scenarios, the absorption energies are strongly dependent on the SND size in unsaturated cases, which implies the importance of the silicon dangling bonds. Our calculation results are also in good agreement with the previous theoretical work on the absorption energies of water on silicence^40^. The calculation results of the absorption energies presented here show the SND is hydrophilic, which is very similar to those for the graphene nanostructure due to the formation of the chemical bonds between silicon/carbon and oxygen atoms. However, one exception is the benzene, which is hydrophobic; this might be due to hydrogen bonds^43^. This aspect needs to be studied carefully further in the future. We can also see that the absorption energy for SND is at least one order larger than that for graphene, which suggests the water molecule is more strongly bonded on the SND than the graphene. Moreover, the calculations for the water absorptions on the $3\times 3$ SND have been performed by adding the dispersion force on top of the B3LYP density functional, as shown in Table 2. The four initial configurations, including top, hollow, valley, and bridge, with the molecule lying on the SND plane and the zero-leg geometry, have been computed. Our calculations therefore show that the top configuration is the most stable for both geometries, which is consistent with the previous work^40^. For the lying down geometry, the absorption energies are slightly different as the optimizations end with different top sites due to the different initial configuration. For the zero-leg geometry, all the optimizations end with the top configuration except the valley one, which can be seen from the larger energy difference (~ 0.1 eV) of the valley configuration from the other initial configurations as shown in Table 2. In addition, compared with the calculations without dispersion forces, the absorption energy per water molecule is slightly lower by ~ 0.1 eV and the bond length between the oxygen atom and the nearest silicon atom decreases by ~ 0.01 angstroms, which is reasonable as the dispersion forces increase the attraction potential. We can also see the tilted angle of the water molecule by ~ 70°. However, there might be complications with the orientations and positions of the water molecule, which need further studies in the future. We have also performed the calculations for the $3\times 3$ SND absorbing a square ice taking into account the Van der Waal forces, which show the absorption energy will be slightly decreased by 0.1 eV per water molecule, which has an insignificant effect on the qualitative picture. A more detailed comparison between the calculations with and without the Van der Waal forces can be performed in the future study.

**Table 2:** Water absorption energies (eV) for lying down and one-leg geometries for different initial attaching positions, including top, hollow, valley, and bridge^40^.

Water molecule geometry/initial configurations	Top	Hollow	Valley	Bridge
Lying down	− 0.47	− 0.46	− 0.48	− 0.46
Zero-leg	− 0.47	− 0.46	− 0.32	− 0.46

a $3\times 3$ SND has been chosen here.

**Figure 7:**
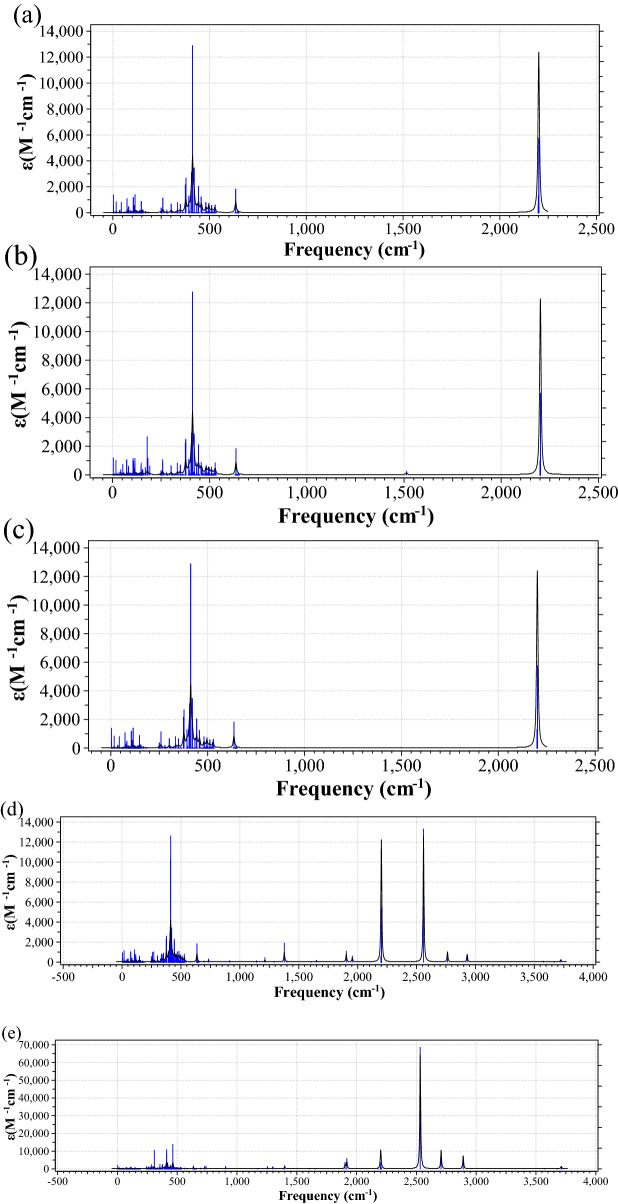
The IR spectra (in the unit of cm^−1^ M^−1^) as a function of vibrational frequencies (in the unit of cm^−1^): (**a**) the $5\times 5$ SND, (**b**) a single water molecule on the centre of the $5\times 5$ SND, (**c**) a single water molecule on the edge of the $5\times 5$ SND, (**d**) a square ice on the centre of the $5\times 5$ SND, and (**e**) a square ice on the edge of the $5\times 5$ SND.

Figure 7a–e show the IR spectra when a single water molecule and a square ice are absorbed on a $5\times 5$ SND at the centre and the edge. The IR spectra are plotted as a function of vibrational frequencies. There are two main peaks at 490 cm^−1^ and 2200 cm^−1^, which is in a good agreement with the recent experimental work on silicene^52^. The 490 cm^−1^ peak involves both the silicon and hydrogen atoms for saturation, whereas the 2200 cm^−1^ peak is dominated by the saturation hydrogen atoms. Compared to Fig. 7b, the presence of a peak at ~ 175 cm^−1^ and the disappearance of a minor peak at ~ 1500 cm^−1^ in Fig. 7c indicates a more complex water absorption result because the hydrogen atoms on the edge of SND are closer to the contact site. Hence, the stretching of the molecule water at the centre gives rise to an additional peak at ~ 175 cm^−1^ in Fig. 7b, which disappeared in Fig. 7c due to the spatial position change of the molecule water. Figure 7d,e are focused on the vibrations of the combined structure of a square ice at the centre and the edge of the silicene to demonstrate the sensitivity of the silicene to the proximity of ice. The difference between these two graphs is that in Fig. 7e there are some less pronounced peaks at 310 cm^−1^ and 470 cm^−1^, with the major difference being the peaks at 2500 cm^−1^, 2700 cm^−1^ and 2900 cm^−1^ producing great increases in value of about five times, which stems predominately from the absorbed water molecule. However, the absorption energy is generally the same for both interaction scenarios, at approximately − 1 eV.

## Conclusions

In summary, we have studied the water contact scenario for SND and pioneered the study of the mechanism of its contact with ice. We have computed the absorption energies of water molecules and square ice on SNDs with three different sizes ($3\times 3$, $4\times 4$, and $5\times 5$) and revealed that the absorption energy is slightly lower at the edges, implying stronger absorption. Our calculation results for the absorption energies are in agreement with the previous work on the similar structures. We have also found that SNDs are hydrophilic and ice-philic both on the centre and the edge of the SND. All the calculations here show that SND is hydrophilic, which is similar to graphene nanostructures except benzene. The water/ice absorption energy of SND is one order larger than those for graphene. The calculations with the Van der Waals forces corrections show that the absorption energies only decrease by ~ 0.1 eV per water molecule. The charge distributions for HOMO and LUMO would tend to be localized on the edge when increasing the size.

Our calculations show that the water molecules and square ice will be tilted on the SND plane each at ~ 70° and ~ 45° respectively, probably as a consequence of the zig-zag structure of the SNDs. Some symmetry might exist between the respective tilts of water and square ice, a feature which is supposed to be directly related to the charge distribution in the contact scenario. Our results have enriched the current wetting mechanism of silicene, which will be relevant for the preparation of 2D silicon-based materials such as the wet chemical separation method, and explored the icing mechanism, which could be used for the semiconducting device applications based on SNDs. The calculation results presented here will facilitate the design of silicene-based electronic devices when taking into account water molecules is necessary.
